# Changes and Correlation Between Hormones, Immunoglobulins, and Minerals in Blood Serum and Hair of Tianzhu White Yaks of Different Ages and Gender

**DOI:** 10.3390/ani15050682

**Published:** 2025-02-26

**Authors:** Yunqi Deng, Bingang Shi, Youpeng Qi, Zhihao Luo, Changze Cui, Shaopeng Chen, Xuelan Zhou, Zhidong Zhao, Xiaolan Zhang, Jiang Hu

**Affiliations:** College of Animal Science and Technology, Gansu Agricultural University, Lanzhou 730070, China; 15002555696@163.com (Y.D.); shibg@gsau.edu.cn (B.S.); qiyp_gsau@163.com (Y.Q.); lzhxxx0104@163.com (Z.L.); cuichangze0120@163.com (C.C.); 15095373670@163.com (S.C.); 14201@gsau.edu.cn (X.Z.); zhaozd@gsau.edu.cn (Z.Z.)

**Keywords:** Tianzhu white yak, hair, hormone, immunoglobulin, mineral element

## Abstract

(1) Background: Hair growth can function as an indicator of an animal’s health and nutritional status, furnishing a valuable means for early diagnosis and timely modifications to management practices, and is influenced by hormones, the immune system, and the levels of minerals within the animal’s body. (2) Methods: In this study, a total of 81 yaks were selected and divided into three groups according to their ages (1–2 years, 3–4 years, 5–6 years), and the concentrations of melatonin, dihydrotestosterone, IgA, IgG, and IgM in their blood serum and the levels of Ca, Mg, Zn, Fe, and P in both their blood serum and hair were detected. (3) Results: The levels of melatonin and dihydrotestosterone in yaks aged 1–2 years were significantly higher than those of other age groups (*p* < 0.001). In addition, the concentration of melatonin in male yaks was markedly greater than that in female yaks (*p* < 0.05). When it came to immunoglobulins, the IgA level in 1–2-year-old yaks was strikingly higher than those in yaks aged 3–4 years and 5–6 years (*p* < 0.05). In terms of mineral elements, the concentrations of calcium, magnesium, zinc, and iron in the blood serum of 1–2-year-old and 5–6-year-old yaks were significantly elevated compared to those in 3–4-year-old yaks. However, there was no notable difference between the 1–2-year-old and 5–6-year-old groups. The correlation analysis showed that there was a significant correlation between DHT and the levels of melatonin and IgA in yaks. Additionally, a significant correlation was observed between IgA and IgG, as well as between IgA and the levels of Fe, Mg, and Ca. It is likely that they jointly regulate the hair growth and immune processes of yaks. (4) Conclusions: There are positive correlations among melatonin, dihydrotestosterone, and IgA in the blood serum and the content of Fe and Mg in the hair to varying degrees, implying these indicators may jointly regulate yak hair growth and immune status. These research findings provide data support for applied research into evaluating the overall health status of yaks through the growth status of hair or by measuring the mineral content in hair in a non-invasive way.

## Simple Summary

This study was designed to probe into the changes in the hormones, immune system proteins, and minerals in the blood and hair of Tianzhu white yaks across different age groups (ranging from 1–2, 3–4, and 5–6 years old) and genders. The results showed that compared with older yaks, younger ones had remarkably higher levels of melatonin and dihydrotestosterone, both of which are crucial for hair growth. Additionally, it was confirmed that the younger yaks contained a greater amount of immune protein IgA, while the levels of IgG and IgM gradually declined as they grew older. In terms of certain minerals in the blood serum, like calcium, magnesium, zinc, and iron, their concentrations were considerably higher in younger and older yaks than in the 3–4-year-old group. In contrast, significant differences in hair minerals were observed among all age ranges, with different change trends for each mineral. Since hair growth is affected by these elements, monitoring them can effectively evaluate the health and nutritional status of yaks. This non-invasive method can assist farmers in closely observing the yaks’ health and making timely adjustments to feeding or treatment plans.

## 1. Introduction

The yak holds an irreplaceable position in the pastoral areas of the Qinghai–Tibet Plateau, providing meat, milk, leather, fur, draft power, etc., and serving as an important material and economic source for local herdsmen. The yak is the only bovine species that can produce velvet, and its velvet-producing characteristics are also the result of its long-term adaptation to the alpine environment. Among them, the hair of the Tianzhu white yak, with its pure white color and excellent dyeability, makes it a more unique and precious raw textile material [1]. Besides the economic value of its hair and down, yak hair, as an important phenotypic trait, is also closely related to the stress resistance, growth, and health of yaks. Hair growth is regulated by a variety of hormones, especially melatonin and androgens, which play important regulatory roles in hair growth [2,3].

Melatonin (MELT) is a hormone mainly produced by the pineal gland and plays a crucial role in regulating various physiological functions in mammals [4]. MELT is an important molecule in animals for regulating circadian rhythms, improving sleep, controlling emotions, stabilizing body temperature, and enhancing immunity [5,6]. MELT is mainly involved in hair pigmentation by increasing the number of melanocytes and also affects hair growth [7]. Mitchell et al. continuously implanted MELT in goats and proved that the implantation accelerated the growth rate of cashmere, indicating that MELT has a certain promoting effect on the growth of down [8]. In addition to affecting hair follicle development, MELT has also been proven to be related to the immune function and antioxidant capacity of the animal body [9,10]. Therefore, combined with the content of the immunoglobulins in the body, the potential regulatory role of MELT on the immune system and the health status of the yak body can be illustrated.

Testosterone (T) is a sex hormone produced by the gonads and adrenal glands. Under normal circumstances, more than 95% of T is irreversibly converted into dihydrotestosterone (DHT) through the action of 5α-reductase [11]. Currently, it is generally believed that T forms DHT under the action of 5α-reductase, thus affecting the growth and development of hair follicles [7]. DHT has been studied in the context of the influence of hormones on hair follicles and, together with T, has been found to play a key role in terminal hair growth [7]. Researchers implanted DHT subcutaneously in mice and observed the growth of their hair. The results showed that DHT has an inhibitory effect on hair regeneration in mice [12]. In humans, studies have also shown that 5α-reductase and DHT mediate the miniaturization of hair follicles and the shortening of successive growth cycles, ultimately leading to androgenetic alopecia [13].

Hair is considered a tissue that is influenced by blood circulation. During the hair growth phase, it absorbs nutrients through the capillaries and interacts with the body’s biological fluids and blood circulation. However, once the hair is keratinized and passes through the epidermis, it no longer contacts these fluids, and the metabolic activities tend to stop. Moreover, compared with other samples such as blood, hair can stably reflect the nutritional status of animals for a long time and has an important application value, especially in long-term monitoring and ecological research [14]. Therefore, theoretically, the mineral concentration in hair can reflect the mineral concentration to which the hair is exposed during hair follicle development and thus can be used as a reliable indicator of the mineral status to measure the mineral element levels in the animal body in a non-invasive way. Mineral elements are essential nutrients for animals and are crucial for maintaining the homeostasis of the animal body, offspring reproduction, and improving production performance [15]. The metabolic requirements of minerals such as calcium (Ca), phosphorus (P), and iron (Fe) vary at different growth stages and are dynamically regulated to meet the needs of bone development, muscle growth, and oxygen transport. Studies have found that the interactions between different minerals further enhance the adaptability of yaks to the plateau environment. For example, Ca and magnesium (Mg) coordinate with each other in maintaining the bone structure [16,17,18], while Fe and zinc (Zn) work together in enhancing the function of the immune system [19]. This multi-level regulation of mineral metabolism not only supports the basic physiological needs of yaks but also enhances their tolerance to the cold and hypoxic environment. The contents of mineral elements in hair and blood serum can not only reflect the nutritional status of the animals but also comprehensively judge the overall health status of the animals in combination with immunoglobulins.

Therefore, by jointly analyzing the correlation between the mineral elements in yak hair and the hormones, immunoglobulins, and mineral elements in the body, it is possible to evaluate the overall health status of yaks through the growth status of hair or by measuring the mineral content of the hair in a non-invasive way.

## 2. Materials and Methods

All animal sampling was approved by the Animal Ethics Committee of Gansu Agricultural University (approval number: GSAU-Eth-AST-2023-014). The animals required for sample collection all came from the herdsmen’s farms (Tianzhu, Gansu Province, China).

### 2.1. Animal Sample Collection

In this study, on 17 June 2024, 81 Tianzhu white yaks were randomly selected in Heimajuan River, Tianzhu Tibetan Autonomous County, Wuwei City, Gansu Province, including 41 male yaks and 40 female yaks. There were 12 yaks aged 1–2 years (5 male yaks and 7 female Yaks), 35 Yaks aged 3–4 years (18 male yaks and 17 female Yaks), and 34 Yaks aged 5–6 years (16 male Yaks and 18 female Yaks). Five milliliters of blood was collected from each yak using jugular vein puncture into two tubes. After the collected blood was shaken well in the anticoagulant tube, it was centrifuged at 3000 r/min for 10 min. The supernatant was taken as blood serum and stored frozen at −20 °C and then brought back to the laboratory. A total of 30 yaks with good hair growth conditions were selected, with 10 yaks from each of the three age groups. About 10 g of the hair samples were cut from the root from the scapular region with scissors. After removing impurities such as soil, the hair was placed in self-sealing bags and awaited laboratory determination.

### 2.2. Determination of MELT, DHT and T

The determination of MELT, DHT, and T was carried out using enzyme-linked immunosorbent assay kits (Beijing Huaying Biotechnology Research Institute, Beijing, China). After processing according to the kit instructions, the blank wells were used to adjust the zero. The absorbance values of each well were measured with a microplate reader (Huaweidelang DR-200BS, Wuxi Huawei Delang Instrument Co., Ltd., Wuxi, China) at a wavelength of 450 nm, and the measurement was completed within 15 min after adding the stop solution. The absorbance values of the standards were taken as the vertical axis, and the corresponding concentrations were taken as the horizontal axis to draw a standard curve. The concentrations of the samples to be tested were looked up on the standard curve.

### 2.3. Determination of Immunoglobulins

The determination of IgG/A/M was carried out using the bovine IgG/A/M kit (HY-755, Beijing Huaying Biotechnology Research Institute, Beijing, China). After operating according to the kit instructions, the measurement was performed using a microplate reader (Huaweidelang DR-200BS). For IgG, A1 was measured at a wavelength of 610 nm, and after incubation at 37 °C for 3–5 min, A2 was measured at a wavelength of 610 nm. For IgA/M, A1 and A2 were measured at a wavelength of 340 nm. Result calculation: The content of IgG/A/M (g/L) = $\frac{∆A\;of\;the\;specimen}{∆A\;of\;the\;standard}\;$ × the concentration of the standard (∆A = A1 − A2).

### 2.4. Determination of Mineral Elements in Blood Serum and Hair

The concentrations of Ca, Mg, Zn, and Fe in the blood serum were determined using an automatic biochemical analyzer (Shenzhen Leidu Life Technology Co., Ltd., Shenzhen, China). The P was determined by colorimetry using a phosphorus kit (Zhong Sheng Bei Kong Biotechnology Co., Ltd., Beijing, China). After pretreatment according to the kit instructions, the blank tube was used to adjust the zero, and the A standard and A sample were measured respectively. Result calculation: $\frac{A\_sample}{A\_standard}$ × standard concentration (mmol/L).

The determination of mineral elements in the hair: 0.1 g of the whole hair cut from the root was weighed, the whole hair was put into a container and to this, 5 mL concentrated HNO_3_ and 1 mL H_2_O_2_ were added, thoroughly mixed, and soaked overnight. The next day, the hair sample was placed on a metal plate and heated at a constant temperature to evaporate the acid until a small amount of residue remained. Then, distilled water was added for redissolution and the total volume was made up to a volume of 1 mL. The sample was left to stand for 1 h and then was measured using an AFS-230 dual-channel atomic fluorescence photometer (Beijing Haiguang Instruments Co., Ltd., Beijing, China). Standard curve: The standard solution was added into a 50 mL volumetric flask, then 10 mL of hydrochloric acid was added to the flask, the corresponding concentration was adjusted with distilled water, and then this was measured on the instrument. The corresponding sample concentration was calculated using the standard curve.

### 2.5. Data Analysis

One-way analysis of variance was used to compare the blood serum hormones, immunoglobulins, and mineral elements among the different age groups as well as the mineral elements in hair. Independent sample *t*-tests were used to analyze and compare the hormones, immunoglobulins, and mineral elements between the different genders. Pearson correlation analysis was used to analyze the correlations between the hormones, immunoglobulins, and mineral elements in the blood serum, and between the mineral elements in the hair.

## 3. Results

### 3.1. Hormone Changes in the Blood Serum of Yaks at Different Ages and Genders

According to Table 1, the MELT and DHT level in yaks aged 1–2 years was significantly higher than those in the aged 3–4 years and 5–6 years groups (*p* < 0.01). Meanwhile, the T concentration was measured. The results showed that the T concentration also tended to decrease with age, but there was no significant difference between the age groups (*p* > 0.05). According to Table 2, for the different genders, MELT concentration in male yaks was significantly higher than that in female yaks (*p* < 0.05). The T content in the blood serum of male yaks was significantly higher than that in female yaks (*p* < 0.01).

### 3.2. Concentration of Immunoglobulins in Yaks of Different Ages and Genders

The results showed that the content of IgA in yaks aged 1–2 years was significantly higher than that in yaks aged 3–4 and 5–6 years (*p* < 0.05), and the contents of IgG and IgM showed a decreasing trend with age, but the difference was not statistically significant (Table 3). There was no significant difference in the content of immunoglobulins between male and female yaks (Table 4).

### 3.3. Concentration of the Mineral Elements in the Hair and Blood Serum of Tianzhu White Yaks at Different Ages and Genders

In the blood serum of Tianzhu white yaks, the levels of two mineral elements, Ca and Mg, were significantly lower in the 3–4-year-old group than those in the 1–2-year-old and 5–6-year-old groups (*p* < 0.01). The content of Zn was significantly higher in the 1–2-year-old and 5–6-year-old groups than that in the 3–4-year-old group (*p* < 0.05), while the level of Fe was significantly lower in the 1–2-year-old and 3–4-year-old groups than that in the 5–6-year-old group (*p* < 0.05), and there was no significant difference in P among the three age groups (Table 5). In the hair, the level of Ca was significantly lower in the 5–6-year-old group than those in the 1–2-year-old and 3–4-year-old groups (*p* < 0.05); the levels of Mg and Fe showed a decreasing trend with age, and there were significant differences among all age groups (*p* < 0.01); the levels of Zn and P were significantly higher in the 3–4-year-old group than those in the 1–2-year-old and 5–6-year-old groups (*p* < 0.01) (Table 6). There was no statistical significance for the mineral elements in the blood serum and hair between different genders.

### 3.4. Correlation Analysis for the Hormones, Immunoglobulins, and Mineral Elements in Tianzhu White Yaks

Correlation analysis revealed that there was a significant positive correlation between MELT and DHT, however, there was no significant correlation between these hormones and T. IgA showed a significant positive correlation with IgG, DHT, Ca, Fe, and Mg. This phenomenon suggests that there might be a synergistic effect among immunoglobulins, DHT, and minerals in the physiological activities of yak blood serum.

**Figure 1:**
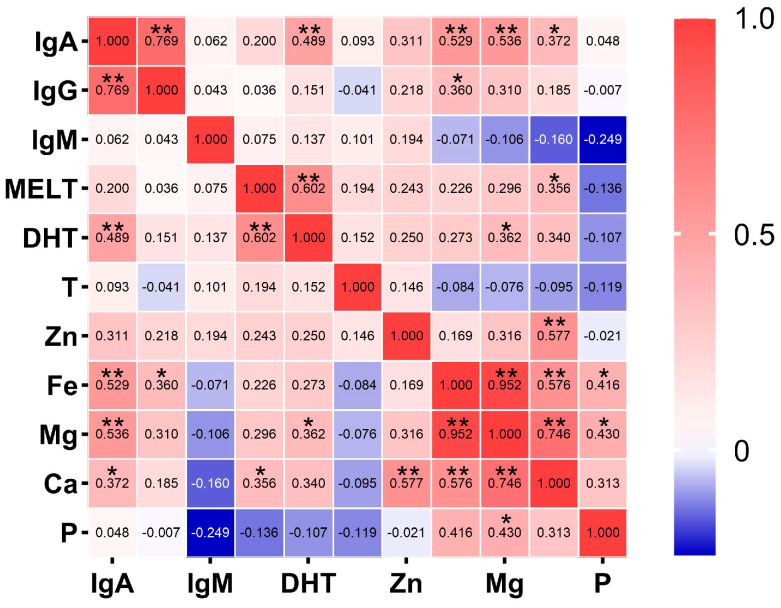
Correlation between the hormones, immunoglobulins, and mineral elements in blood serum. * indicates *p* < 0.05, and ** indicates *p* < 0.01. The vertical axis from top to bottom represent immunoglobulin A, immunoglobulin G, immunoglobulin M, melatonin, dihydrotestosterone, testosterone, zinc, iron, magnesium, calcium, and phosphorus, respectively. The horizontal axis from left to right each column of immunoglobulin A, immunoglobulin G, immunoglobulin M, melatonin, dihydrotestosterone, testosterone, zinc, iron, magnesium, calcium, phosphorus. Because it would be too crowded to display all the horizontal axes, one name was displayed every other column.

Regarding the mineral elements, a significant positive correlation was found between Mg and Ca, Fe, and P, which illustrates that the mineral elements coordinate with each other to meet the normal growth and development requirements of the body. Ca also had a significant positive correlation with MELT, Zn, Fe, and Mg. The correlation between Ca and MELT further demonstrated that MELT likely plays a positive role in addressing calcium deficiency, and Ca also influences the secretion of MELT (Figure 1).

**Figure 2:**
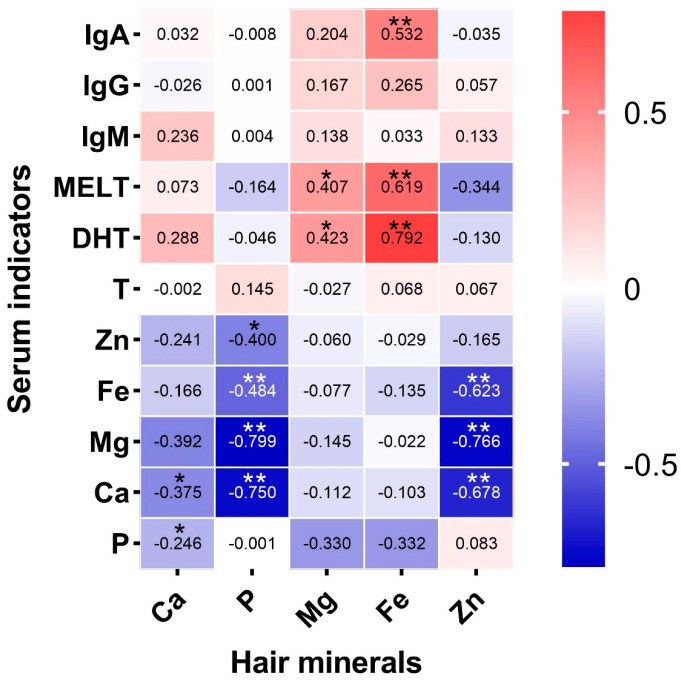
Correlation between the hair minerals and blood serum hormones, immunoglobulins, mineral elements, * indicates *p* < 0.05, ** indicates *p* < 0.01. From left to right, the horizontal axis shows the minerals calcium, phosphorus, magnesium, iron, and zinc in hair, respectively. From top to bottom, the vertical axis shows the blood serum immunoglobulin A, immunoglobulin G, immunoglobulin M, melatonin, dihydrotestosterone, testosterone, zinc, iron, magnesium, calcium, and phosphorus, respectively.

Moreover, from the whole group, 30 yaks were selected to analyze the correlation between hair minerals and serum indexes; in the analysis of the correlation between the hair minerals and blood serum indicators, Fe in hair had a significant positive correlation with IgA, MELT, and DHT, and Mg also exhibited a significant positive correlation with MELT and DHT. Meanwhile, Ca, P, and Zn in hair had a significant negative correlation with multiple elements in the blood serum. Such a negative correlation helps us establish a dynamic monitoring model for the mineral element content in hair and blood serum (Figure 2).

## 4. Discussion

In mammals, hair has the functions of protection and evolution. Having healthy hair also represents that the animal individual is healthy, young, and energetic [20]. The hair follicle is an important organ for producing hair. According to previous studies, sex hormones have a negative impact on the hair follicle, miniaturizing the terminal hair follicle, and ultimately leading to the transformation into vellus hair or atrophied vellus hair [21]. Other researchers observed the hair growth results of androgenetic alopecia patients after using MELT. The patients using MELT had improved hair growth, density, and hair shaft thickness, proving that MELT has a positive promoting effect on the hair follicle and hair growth [22]. In the results of this study, both MELT and DHT showed a decreasing trend with age, and they had a positive correlation. This is because the high level of DHT in the juvenile stage can promote muscle and bone development. The effect of this hormone enhances the adaptability of the yaks to the low-temperature and hypoxic environment, enhancing the cold tolerance and stress resistance of juvenile yaks [23]. At the same time, in order to ensure the normal growth of hair, yaks will not have poor hair follicle development or hair loss due to the high level of DHT. MELT also needs to be at a high level in the body to inhibit the negative effect of DHT on hair follicle growth and development and ensure the normal growth of hair. Therefore, the changes in concentration MELT and DHT are always consistent. The high level of MELT can also improve antioxidant capacity and effectively resist the strong ultraviolet radiation on the Qinghai–Tibet Plateau, thereby reducing the oxidative stress caused by the environment [24].

Immunoglobulins play a crucial immune function in the animal body and mainly include types such as IgA, IgG, and IgM. Immunoglobulins are the core components of the body’s immune system. They provide a defense mechanism for animals by recognizing and neutralizing pathogens (such as bacteria and viruses) [25]. The content level of immunoglobulins is often used as an important indicator to measure the strength of the body’s immune function. Generally speaking, the higher the concentration of immunoglobulins, the stronger the immune function of the body and the stronger the ability to resist external pathogens [26]. Recent studies have shown that MELT is not only a hormone that regulates sleep but also has significant immunomodulatory and anti-inflammatory effects [10]. MELT promotes the balance of the immune response and enhances the body’s resistance by interacting with the body’s immune system, especially in dealing with pathogen infection and inflammatory responses [27]. Some studies have shown that alopecia is an autoimmune disease caused by the immune system attacking the hair follicle, and regulating this immune response can promote hair regeneration [28]. Other research reports have also found that there is lymphocyte infiltration and complement C3 and immunoglobulin deposition around the hair follicles in the alopecia area, indicating that alopecia is related to the inflammatory immune response [29]. The above research results all indicate that the immune response is related to the growth and apoptosis of the hair follicle, and as immunoglobulins are part of the body’s immune system, the content of immunoglobulins is also related to the growth and development of hair. In this study, it was observed that the content of immunoglobulins showed a decreasing trend with age, which was highly consistent with the changes in the MELT level. Specifically, the concentration of immunoglobulins gradually decreased with age. This phenomenon is particularly important in animal husbandry, especially in the process of yak breeding. Yaks need a higher level of immunoglobulins in the juvenile stage to enhance their disease resistance and ensure their survival rate. The juvenile stage is a critical stage for the development and maturation of the immune system. Therefore, at this stage, a higher level of immunoglobulins can effectively resist pathogen invasion and enhance the body’s immune response. However, as the age of the yaks increases, the immune system gradually develops and matures, and the immune function tends to become stable. The demand for immunoglobulins gradually decreases and the immunoglobulins can effectively play an immune role by maintaining a certain level. In addition, the timely supplementation of immunomodulators such as MELT can effectively improve the immunity of yaks, reduce the occurrence of diseases, improve hair growth conditions, and thus improve the breeding efficiency.

Hair is one of the indicators that reflect the nutritional status of various mineral elements in the animal body. The mineral content in hair can reflect the nutritional status of the animals within a certain period, and the deficiency or excess of various mineral elements is often related to disease. The content of minerals in hair may be helpful as an early diagnosis tool for many diseases [30]. From the results of this study, the change trends of the different mineral elements in the blood serum and hair were different at different age stages. In the plateau environment, the demand for minerals significantly increases to support the development of physiological structures and functions. The concentration fluctuations of calcium and magnesium at different age stages are particularly significant, indicating that they jointly support bone density and muscle function [31], and their metabolisms have a positive correlation. This metabolic interaction indicates that Tianzhu white yaks have a higher demand for calcium and magnesium intake at specific growth stages to support the development of bones and muscles. Zinc and iron play important roles in hematopoiesis, immune enhancement, and antioxidant stress [32]. This mineral metabolism pattern may be an adaptive manifestation of Tianzhu white yaks in the plateau environment to deal with external oxidative stress, helping them maintain bone strength and muscle function. P plays a central role in cell signal transduction, energy metabolism, and bone development [33], and it also acts as a feedback regulator for immune system activity [34]. In this study, P showed a negative correlation with IgG and IgM (*p* > 0.05), which may be due to phosphorus potentially inhibiting the excessive secretion of immunoglobulins to optimize resource allocation in the body. This mechanism may prioritize metabolic and skeletal growth needs when necessary, ensuring normal hair growth while maintaining the ability to clear pathogens. Such a balanced mechanism may help yaks better survive and reproduce in the extreme environment of the Tibetan Plateau.

## 5. Conclusions

This study investigated the relationship between the hormones, immunoglobulins, and mineral levels in the blood and hair of Tianzhu white yaks of different ages and genders. The results show significant changes in hormone levels (MELT and DHT), IgA, and mineral content (Ca, Mg, Zn, Fe) as the yaks age. Younger yaks exhibited higher levels of MELT and DHT, which are critical for hair growth. Additionally, the IgA levels in younger yaks were significantly higher than those in the other two age groups, while IgG and IgM levels decreased gradually with age. In terms of blood mineral content, calcium, magnesium, zinc, and iron were found to be higher in both younger and older yaks compared to the middle-aged group (3–4 years), with distinct patterns observed in the mineral content of the hair. The correlation analysis indicated positive relationships between MELT, DHT, IgA, and the levels of iron and magnesium in the hair, suggesting that these indicators may collectively regulate hair growth and immune function in yaks. These findings provide valuable insights into the use of non-invasive methods, such as analyzing hair mineral content, to monitor the health and nutritional status of yaks. This approach can aid in early disease diagnosis, treatment, or adjustments to feeding practices.

## Figures and Tables

**Table 1:** Concentrations of MELT, DHT, and T in white yaks at different ages.

Hormone	Ages			*p*
	1~2 Years Old	3~4 Years Old	5~6 Years Old	
MELT, pg/mL	60.36 ± 2.13 ^A^	49.18 ± 1.97 ^B^	44.16 ± 1.48 ^B^	<0.0001
DHT, pg/mL	316.8 ± 11.60 ^A^	229.5 ± 5.16 ^B^	199.2 ± 5.69 ^C^	<0.0001
T, pg/mL	476.2 ± 158.4	425.1 ± 51.1	353.6 ± 61.7	0.5542

The data in the table are presented as mean ± SEM. Different uppercase superscript letters indicate extremely significant differences between groups at the *p* < 0.01 level, and no letter or the same letter indicates no significant difference between groups. MELT stands for melatonin, DHT stands for dihydrotestosterone, and T stands for testosterone.

**Table 2:** Concentrations of MELT, DHT and T in Tianzhu white yak of different genders.

Hormone	Gender		*p*
	Male	Female	
MELT, pg/mL	51.64 ± 1.77 ^a^	45.749 ± 1.63 ^b^	0.0167
DHT, pg/mL	237.4 ± 8.67	221.8 ± 7.19	0.1686
T, pg/mL	544.5 ± 63.72 ^A^	257.3 ± 40.8 ^B^	0.0003

The data in the table are presented as mean ± SEM. Different lowercase superscript letters indicate significant differences between groups at the *p* < 0.05 level, different uppercase superscript letters indicate extremely significant differences between groups at the *p* < 0.01 level, and no letter or the same letter indicates no significant difference between groups. MELT stands for melatonin, DHT stands for dihydrotestosterone, and T stands for testosterone.

**Table 3:** Immunoglobulin concentrations of white yaks at different ages.

Immunoglobulins	Ages			*p*
	1~2 Years Old	3~4 Years Old	5~6 Years Old	
IgA, g/L	2.181 ± 0.08 ^A^	1.947 ± 0.03 ^B^	1.814 ± 0.06 ^B^	0.0030
IgG, g/L	8.849 ± 0.41	8.348 ± 0.19	8.169 ± 0.39	0.4343
IgM, g/L	0.6580 ± 0.03	0.6575 ± 0.01	0.6332 ± 0.03	0.7079

The data in the table are presented as mean ± SEM. Different uppercase superscript letters indicate extremely significant differences between groups at the *p* < 0.01 level, and no letter or the same letter indicates no significant difference between groups. IgA represents immunoglobulin A, IgG represents immunoglobulin G, and IgM represents immunoglobulin M.

**Table 4:** Immunoglobulin concentrations of white yaks at different gender.

Immunoglobulins	Gender		*p*
	Male	Female	
IgA, g/L	1.917 ± 0.06	1.966 ± 0.04	0.7229
IgG, g/L	8.165 ± 0.31	8.602 ± 0.21	0.2567
IgM, g/L	0.6395 ± 0.02	0.6568 ± 0.02	0.5488

The data in the table are presented as mean ± SEM. No letter indicates no significant difference between groups. IgA represents immunoglobulin A, IgG represents immunoglobulin G, and IgM represents immunoglobulin M.

**Table 5:** Concentration of mineral elements in the blood serum of white yaks at different ages.

Mineral	Ages			*p*
	1~2 Years Old	3~4 Years Old	5~6 Years Old	
Ca, mmol/L	1.507 ± 0.090 ^A^	0.617 ± 0.024 ^B^	1.647 ± 0.300 ^A^	<0.0001
Mg, mmol/L	1.601 ± 0.046 ^A^	0.630 ± 0.017 ^B^	1.569 ± 0.067 ^A^	<0.0001
Zn, umol/L	33.87 ± 3.729 ^a^	21.70 ± 2.058 ^b^	35.33 ± 3.750 ^a^	0.0140
Fe, umol/L	30.96 ± 3.915 ^a^	20.60 ± 3.189 ^a^	36.82 ± 3.590 ^b^	0.0136
P, ug/L	56.91 ± 3.620	64.58 ± 3.733	69.82 ± 5.865	0.1346

Data in the table are means ± SEM, different lowercase letters indicate significant differences between groups at the *p* < 0.05 level, different uppercase letters indicate very significant differences between groups at the *p* < 0.01 level, and no letters or the same letters indicate no significant differences between groups. Ca, Mg, Zn, Fe, and P refer to the mineral elements calcium, magnesium, zinc, iron, and phosphorus, respectively.

**Table 6:** Concentration of mineral elements in the hair of white yak at different ages.

Mineral	Ages			*p*
	1~2 Years Old	3~4 Years Old	5~6 Years Old	
Ca, ug/g	1957 ± 151.5 ^a^	2043 ± 161.6 ^a^	1453 ± 83.9 ^b^	0.0104
Mg, ug/g	357.7 ± 33.01 ^A^	314.4 ± 21.14 ^A^	226.0 ± 21.01 ^B^	0.0040
Zn, ug/g	100.9 ± 5.34 ^B^	151.5 ± 5.67 ^A^	103.4 ± 6.08 ^B^	<0.0001
Fe, ug/g	234.4 ± 6.5 ^A^	187.5 ± 8.7 ^B^	137.1 ± 6.1 ^C^	<0.0001
P, ug/g	69.73 ± 3.45 ^B^	97.57 ± 3.96 ^A^	63.55 ± 2.18 ^B^	<0.0001

The data in the table are presented as mean ± SEM. Different lowercase superscript letters indicate significant differences between groups at the *p* < 0.05 level, different uppercase superscript letters indicate extremely significant differences between groups at the *p* < 0.01 level, and no letter or the same letter indicates no significant difference between groups. Ca, Mg, Zn, Fe, and P refer to the mineral elements calcium, magnesium, zinc, iron, and phosphorus, respectively.

## Data Availability

Data are contained within the article.

## References

[1] Mo L., Ma J., Xiong Y., Xiong X., Lan D., Li J., Yin S. (2023). Factors Influencing the Maturation and Developmental Competence of Yak (*Bos grunniens*) Oocytes In Vitro. Genes.

[2] Song L., Cui Y., Xiao L., Yu S., He J. (2020). DHT and E2 synthesis-related proteins and receptors expression in male yak skin during different hair follicle stages. Gen. Comp. Endocrinol..

[3] McCloghry E., Foldes A., Hollis D., Rintoul A., Maxwell C., Downing J., Baker P., Kennedy J., Wynn P. (1992). Effects of pinealectomy on wool growth and wool follicle density in Merino sheep. J. Pineal Res..

[4] Claustrat B., Leston J. (2015). Melatonin: Physiological effects in humans. Neurochirurgie.

[5] Galano A., Tan D.X., Reiter R.J. (2011). Melatonin as a natural ally against oxidative stress: A physicochemical examination. J. Pineal Res..

[6] Calvo J.R., González-Yanes C., Maldonado M.D. (2013). The role of melatonin in the cells of the innate immunity: A review. J. Pineal Res..

[7] Grymowicz M., Rudnicka E., Podfigurna A., Napierala P., Smolarczyk R., Smolarczyk K., Meczekalski B. (2020). Hormonal Effects on Hair Follicles. Int. J. Mol. Sci..

[8] Mitchell R.J., Betteridge K., Gurnsey M.P., Welch R.A.S. (1991). Fibre growth of cashmere-bearing goats given melatonin in late autumn and winter. N. Z. J. Agric. Res..

[9] Gasmi A., Shanaida M., Oleshchuk O., Semenova Y., Mujawdiya P.K., Ivankiv Y., Pokryshko O., Noor S., Piscopo S., Adamiv S. (2023). Natural Ingredients to Improve Immunity. Pharmaceuticals.

[10] Minich D.M., Henning M., Darley C., Fahoum M., Schuler C.B., Frame J. (2022). Is Melatonin the “Next Vitamin D”?: A Review of Emerging Science, Clinical Uses, Safety, and Dietary Supplements. Nutrients.

[11] Frick J., Aulitzky W. (1991). Physiology of the prostate. Infection.

[12] Naito A., Sato T., Matsumoto T., Takeyama K., Yoshino T., Kato S., Ohdera M. (2008). Dihydrotestosterone inhibits murine hair growth via the androgen receptor. Br. J. Dermatol..

[13] Nestor M.S., Ablon G., Gade A., Han H., Fischer D.L. (2021). Treatment options for androgenetic alopecia: Efficacy, side effects, compliance, financial considerations, and ethics. J. Cosmet. Dermatol..

[14] Stoklasová L., Váhala J., Hejcmanová P. (2019). Minerals in the Blood, Hair, and Faeces of the Critically Endangered Western Derby Eland Under Human Care in Two Wildlife Reserves in Senegal. Biol. Trace Element Res..

[15] Torres C.A., Korver D.R. (2018). Influences of trace mineral nutrition and maternal flock age on broiler embryo bone development. Poult. Sci..

[16] Sayiner S., Fidanci U.R., Kucukersan S., Kismali G., Meral O., Sehirli A.O., Sel T., Karagul H. (2020). Vitamin A, calcium, phosphorus and magnesium status of heifers grazing in Northern Cyprus. Trop. Anim. Health Prod..

[17] Georgieff M.K. (2020). Iron deficiency in pregnancy. Am. J. Obstet. Gynecol..

[18] Chen Z., Zhang W., Wang M., Backman L.J., Chen J. (2022). Effects of Zinc, Magnesium, and Iron Ions on Bone Tissue Engineering. ACS Biomater. Sci. Eng..

[19] Maggini S., Pierre A., Calder P.C. (2018). Immune Function and Micronutrient Requirements Change over the Life Course. Nutrients.

[20] Park A.M., Khan S., Rawnsley J. (2018). Hair Biology: Growth and Pigmentation. Facial Plast. Surg. Clin. N. Am..

[21] Cuevas-Diaz Duran R., Martinez-Ledesma E., Garcia-Garcia M., Bajo Gauzin D., Sarro-Ramírez A., Gonzalez-Carrillo C., Rodríguez-Sardin D., Fuentes A., Cardenas-Lopez A. (2024). The Biology and Genomics of Human Hair Follicles: A Focus on Androgenetic Alopecia. Int. J. Mol. Sci..

[22] Babadjouni A., Reddy M., Zhang R., Raffi J., Phong C., Mesinkovska N. (2023). Melatonin and the Human Hair Follicle. J. Drugs Dermatol..

[23] Vandenput L., Boonen S., Van Herck E., Swinnen J.V., Bouillon R., Vanderschueren D. (2002). Evidence from the aged orchidectomized male rat model that 17beta-estradiol is a more effective bone-sparing and anabolic agent than 5alpha-dihydrotestosterone. J. Bone Miner. Res..

[24] Reiter R.J., Mayo J.C., Tan D., Sainz R.M., Alatorre-Jimenez M., Qin L. (2016). Melatonin as an antioxidant: Under promises but over delivers. J. Pineal Res..

[25] Megha K., Mohanan P. (2021). Role of immunoglobulin and antibodies in disease management. Int. J. Biol. Macromol..

[26] Seria E., Tagliaferro S.S., Cutajar D., Galdies R., Felice A. (2021). Immunoglobulin G in Platelet-Derived Wound Healing Factors. BioMed Res. Int..

[27] Li Y., Yang Y., Li S., Ye Y., Du X., Liu X., Jiang Q., Che X. (2023). Effects of dietary melatonin on antioxidant and immune function of the Pacific white shrimp (*Litopenaeus vannamei*), as determined by transcriptomic analysis. Comp. Biochem. Physiol. D-Genom. Proteom..

[28] Pratt C.H., King L.E., Messenger A.G., Christiano A.M., Sundberg J.P. (2017). Alopecia areata. Nat. Rev. Dis. Primers.

[29] Igarashi R., Takeuchi S., Sato Y. (1980). Immunofluorescent studies of complement C3 in the hair follicles of normal scalp and of scalp affected by alopecia areata. Acta Dermato-Venereol..

[30] Wołowiec P., Michalak I., Chojnacka K., Mikulewicz M. (2013). Hair analysis in health assessment. Clin. Chim. Acta.

[31] Ciosek Ż., Kot K., Kosik-Bogacka D., Łanocha-Arendarczyk N., Rotter I. (2021). The Effects of Calcium, Magnesium, Phosphorus, Fluoride, and Lead on Bone Tissue. Biomolecules.

[32] Stiles L.I., Ferrao K., Mehta K.J. (2024). Role of zinc in health and disease. Clin. Exp. Med..

[33] Goebel K.P., Stein H.H. (2011). Phosphorus digestibility and energy concentration of enzyme-treated and conventional soybean meal fed to weanling pigs1. J. Anim. Sci..

[34] Chen K., Jiang W.-D., Wu P., Liu Y., Kuang S., Tang L., Tang W., Zhang Y.-A., Zhou X.-Q., Feng L. (2017). Effect of dietary phosphorus deficiency on the growth, immune function and structural integrity of head kidney, spleen and skin in young grass carp (*Ctenopharyngodon idella*). Fish Shellfish. Immunol..

